# How ‘omics technologies can drive plant engineering, ecosystem surveillance, human and animal health

**DOI:** 10.1042/ETLS20220020

**Published:** 2022-04-11

**Authors:** Bhavna Hurgobin, Mathew G. Lewsey

**Affiliations:** 1 La Trobe Institute for Agriculture and Food, La Trobe University, AgriBio Building, Bundoora, VIC 3086, Australia; 2 Australian Research Council Research Hub for Medicinal Agriculture, La Trobe University, AgriBio Building, Bundoora, VIC 3086, Australia

**Keywords:** big data, genomics, metabolomics, proteomics, systems biology, transcriptomics

## Abstract

‘Omics describes a broad collection of research tools and techniques that enable researchers to collect data about biological systems at a very large, or near-complete, scale. These include sequencing of individual and community genomes (genomics, metagenomics), characterization and quantification of gene expression (transcriptomics), metabolite abundance (metabolomics), protein content (proteomics) and phosphorylation (phospho-proteomics), amongst many others. Though initially exploited as tools for fundamental discovery, ‘omics techniques are now used extensively in applied and translational research, for example in plant and animal breeding, biomarker development and drug discovery. In this collection of reviews, we aimed to introduce readers to current and future applications of ‘omics technologies to solve real-world problems.

Plant breeders, geneticists and biotechnologists have been enthusiastic adopters of ‘omics approaches. High-throughput short-read and long-read sequencing techniques have enabled the establishment of reference genome sequences and comprehensive gene annotations for many important crops, including those with relatively large or complex genomes [1–4]. This has greatly empowered approaches for defining the genetics underlying crop traits. In a suite of three reviews, authors in this issue discuss how state-of-the-art ‘omics techniques can further enhance plant improvement efforts. Epigenomics is an assembly of information layered on top of DNA sequence that influences gene expression and genome properties. The article of Zhang and colleagues considers how features such as DNA methylation, chromatin status and modifications might be used for precision engineering of crops, such as grains and horticultural plants [5]. Plants produce tens or hundreds of specialized metabolites, which differ widely between species and have potential applications in food, nutrition or health. To do so clear understanding of the biochemical pathways underlying these metabolites is needed, but for most this remains unknown. Muhich and colleagues review how multi-omics tools might be used to overcome this limitation and drive new applications of specialized metabolites in synthetic biology and crop engineering [6]. Lastly, we discuss the need to study plants at tissue-specific and single-cell resolution [7]. Given that most developmental and metabolic processes are highly spatially and temporally localized, this knowledge will allow us to precisely modify plant function and better exploit crop potential.

‘Omics technologies can generate extremely large, rich datasets that are frequently used to study single organisms in great depth. However, they can also be used to look at systems in a very broad way, to better understand the complex interactions between many organisms. An example is the investigation of plant-microbe interactions, which may encompass a single plant species but hundreds or thousands of different fungi, bacteria, protozoa and other microorganisms. As reviewed by Gupta and colleagues, metabolomics has emerged as a very insightful tool to define the physiological and mechanistic properties of these communities [8]. The article details the latest workflows and experimental setups that can be used in such studies, considering both their prospects and limitations. Scaling up even further, ‘omics technologies can be used to monitor function and health at ecosystem-scale. Beale and colleagues propose that multi-omics strategies — being the application of several different ‘omics technologies to a single question — are an effective tool for environmental management and ecosurveillance [9]. This arises from the ability of the technologies to collect data in an untargeted manner, which gives a broader representation of the ecosystem than traditional, targeted approaches.

The final pair of reviews in this issue discuss the applications of ‘omics technologies in human and animal health. Milk and meat derived from cattle is a large component of human diets in many countries. Many different traits must be considered by cattle breeders and producers when managing herds, such as yield, quality, resistance to disease and methane emission. Wang and Guan describe how, in recent years, the cattle microbiome has been demonstrated to significantly influence these traits [10]. Initial research focused on characterizing which microbes are present using metagenomics and related methods, but determining causal relationships with traits from such studies was challenging. The authors critically discuss how multi-omics strategies might be applied to generate functional understanding of how the microbiome of beef and dairy cattle affects production traits, and how manipulation of the microbiome might be used to enhance those traits. Finally, Ahmed considers how precision medicine driven by ‘omics data can be used by clinicians to improve patient healthcare outcomes [11]. The ever-decreasing cost and difficulty of ‘omics analyses enables the generation of comprehensive information about individual patients which can then be combined with clinical data. This allows medical practitioners to make better informed decisions about likely disease progression and optimal treatment strategies. The review also considers the importance of taking a FAIR approach (Findable, Accessible, Intelligent and Reproducible) to enhance the uptake of precision medicine going forward.

In assembling this special issue, we aimed to include a range of different organisms, systems and techniques. We hope that readers find the articles informative with respect to both the fundamentals of technologies and the significant translational impact ‘omics approaches already have across many different domains.

## Open Access

Open access for this article was enabled by the participation of La Trobe University in an all-inclusive *Read & Publish* agreement with Portland Press and the Biochemical Society under a transformative agreement with CAUL.
